# Supplementary material for: Causal interplay between lactose intolerance and gut microbiota: a combined bidirectional Mendelian randomization and in vivo validation study
Source: Front Nutr. 2026 Jun 1;13:1803337. doi: 10.3389/fnut.2026.1803337 (PMC13265576; doi:10.3389/fnut.2026.1803337)
Supplement: Supplementary file 1 [file Data_Sheet_1.zip › supplementary materials/Forward/leave one out cross validation/ebi-a-GCST90027543.finngen_R12_E4_LACTONAS.pdf]

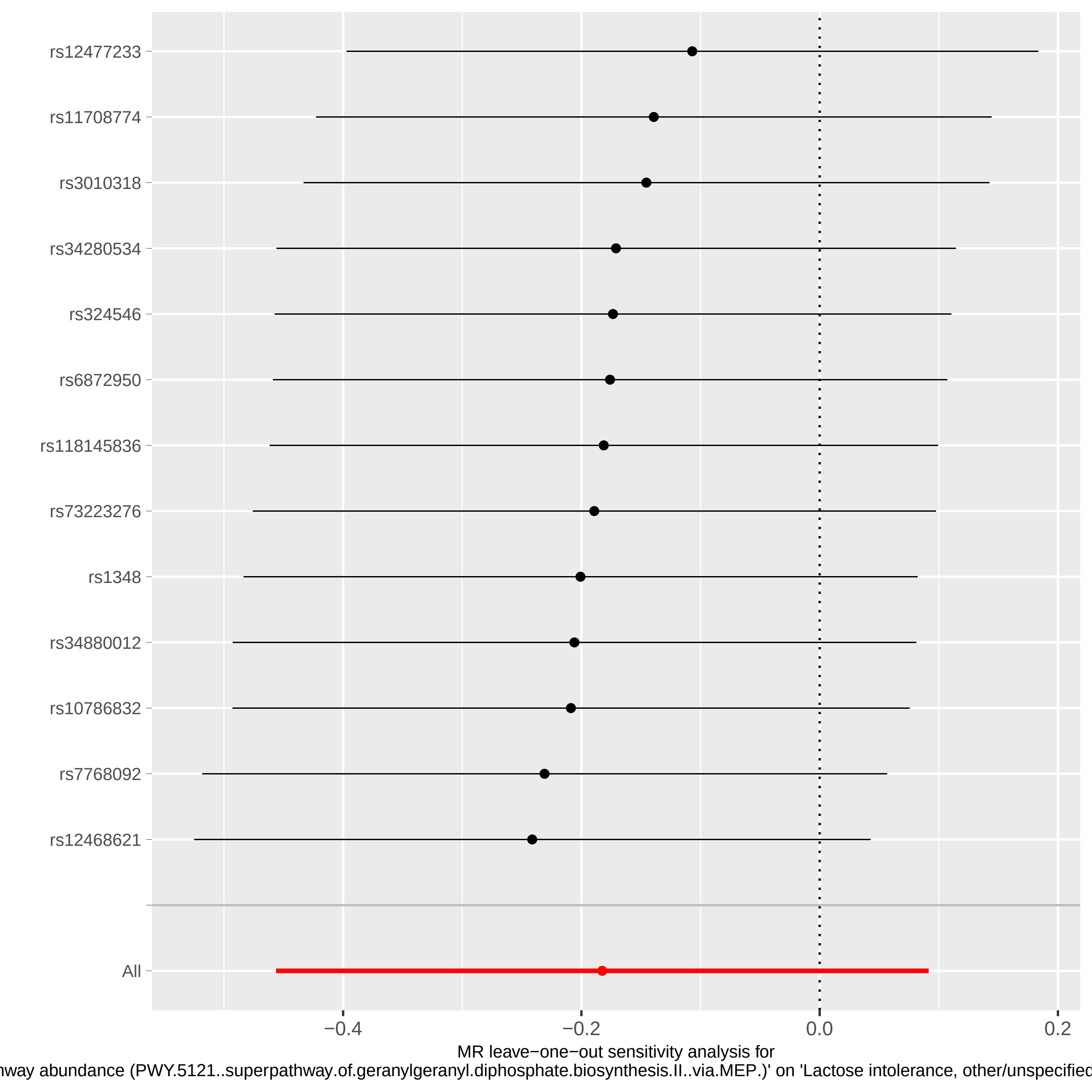

rs12477233
rs11708774
rs3010318
rs34280534
rs324546
rs6872950
rs118145836
rs73223276
rs1348
rs34880012
rs10786832
rs7768092
rs12468621
All
−0.4
−0.2
0.0
0.2
MR leave−one−out sensitivity analysis for
hway abundance (PWY.5121..superpathway.of.geranylgeranyl.diphosphate.biosynthesis.II..via.MEP.)' on 'Lactose intolerance, other/unspecified
